# Are Nutritional Guidelines Followed in the Pediatric Intensive Care Unit?

**DOI:** 10.3389/fped.2021.648867

**Published:** 2021-06-07

**Authors:** Mylène Jouancastay, Camille Guillot, François Machuron, Alain Duhamel, Jean-Benoit Baudelet, Stéphane Leteurtre, Morgan Recher

**Affiliations:** ^1^Pediatric Intensive Care Unit, CHU Lille, Lille, France; ^2^Department of Methodology, Biostatistics, and Management, CHU Lille, Lille, France; ^3^ULR 2694 - METRICS: Évaluation des Technologies de Santé et des Pratiques Médicales, Univ. Lille, CHU Lille, Lille, France; ^4^Congenital and Pediatric Cardiology Unit, CHU Lille, Lille, France

**Keywords:** guidelines, malnutrition, enteral nutrition, energy intake, pediatric intensive care

## Abstract

**Background:** French (2014) and American (2017) pediatric guidelines recommend starting enteral nutrition (EN) early in pediatric intensive care. The aims of this study were to compare the applicability of the guidelines in the pediatric intensive care unit (PICU) and to identify risk factors of non-application of the guidelines.

**Methods:** This retrospective, single-center study was conducted in a medical–surgical PICU between 2014 and 2016. All patients from 1 month to 18 years old with a length of stay >48 h and an exclusive EN at least 1 day during the PICU stay were included. The outcome variable was application of the 2014 and 2017 guidelines, defined by energy intakes ≥90% of the recommended intake at least 1 day as defined by both guidelines. The risk factors of non-application were studied comparing “optimal EN” vs. “non-optimal EN” groups for both guidelines.

**Results:** In total, 416 children were included (mortality rate, 8%). Malnutrition occurred in 36% of cases. The mean energy intake was 34 ± 30.3 kcal kg^−1^ day^−1^. The 2014 and 2017 guidelines were applied in 183 (44%) and 296 (71%) patients, respectively (*p* < 0.05). Following the 2017 guidelines, enteral energy intakes were considered as “satisfactory enteral intake” for 335 patients (81%). Hemodynamic failure was a risk factor of the non-application of both guidelines.

**Conclusion:** In our PICU, the received energy intake approached the level of intake recommended by the American 2017 guidelines, which used the predictive Schofield equations and seem more useful and applicable than the higher recommendations of the 2014 guidelines. Multicenter studies to validate the pediatric guidelines seem necessary.

## What Is Known

- Malnutrition is frequent and a source of high morbi-mortality in the pediatric intensive care unit (PICU).
- Early adequate nutritional therapy is recommended.
- The application of recent pediatric nutritional guidelines has not been previously studied.

## What Is New

We found that energy intake in the PICU approached the intake recommended by the American 2017 guidelines, which used the predictive Schofield equations and seem more useful and applicable than the higher recommendations of the 2014 guidelines.

## Introduction

The prevalence of malnutrition at pediatric intensive care unit (PICU) admission is high (30–50%) and associated with a longer period of mechanical ventilation and higher rates of mortality and nosocomial infections (1–4). In the PICU, oral nutrition was most often impossible. The European Society for Paediatric Gastroenterology Hepatology and Nutrition (ESPGHAN) suggested the use of enteral nutrition (EN) support in critically ill children (5). However, the evaluation of the nutritional status and optimal energy requirements for children in the PICU remains challenging. Early (first 24–48 h) EN is recommended, and optimal nutrition should be attained at the end of the first week of hospitalization (6, 7). If indirect calorimeter was not available, energy requirements can be estimated in the PICU using predicted equations or nutritional guidelines based on healthy children (8, 9).

Several previous studies have shown that the levels of energy intake in the PICU were less than those estimated by equations and have identified risk factors of the non-application of nutritional guidelines (10–12). In the past few years, the French Society of Anesthesia and Intensive Care Medicine (2014) and the American Pediatric Nutrition Group (2017) published guidelines for nutrition support in the PICU (6, 7). However, no study has evaluated or compared the applicability of these two guidelines in the PICU.

The aims of the present study were to (1) compare the applicability of the 2014 and 2017 guidelines and (2) identify risk factors of the non-application of both these guidelines in the PICU.

## Methods

This retrospective, single-center study was conducted in a medical–surgical PICU between October 2014 and December 2016. All patients aged from 1 month to 18 years with a length of stay >48 h and an exclusive EN for at least 1 day during the PICU stay were included. The exclusion criteria were: (a) patients with PICU length of stay <48 h; (b) no exclusive EN during the PICU stay; (c) oral nutrition exclusively; (d) parenteral nutrition exclusively; (e) contraindication to EN (digestive surgery, occlusive syndrome, and bowel ischemia); and (f) missing data.

The data collected (ICCA, Philips^®^) were: diagnosis at admission, Pediatric Index of Mortality 2 (PIM2) score, hemodynamic failure (defined by use of vasopressor agents), invasive or non-invasive ventilation, sedation (defined by use of hypnotic and morphinic agents), paralytic agents, length of PICU and hospital stay, and mortality at 60 days (D60). The daily EN energy intake was calculated during the first 10 days in PICU (considered as the acute phase in our study). The following EN data were collected: time to initiate, volume (in milliliters per hour), energy (in calories per milliliter), interruption and feeding intolerance (defined by vomiting or diarrhea as more than three liquid stools per day), and constipation (need for a laxative or intrarectal treatment).

Nutritional status was determined using the *z*-score weighted for age (13). Malnutrition included underweight (*z*-score <-2) and overweight (*z*-score >2). Malnourished and normo-nourished patients were compared.

The energy requirements differed between the two guidelines (Supplementary Table 1) (6, 7). Firstly, for each patient, both guidelines were considered as “applied” if the patient received more than 90% of the recommended energy intake, considering his age classification, at least 1 day during the hospitalization (10). Secondly, energy intakes were considered as “satisfactory enteral intake” if they were ≥60% of the energy recommended by the 2017 guidelines by the end of the PICU stay (maximum 1 week) (7).

Risk factors of the non-application of the guidelines were studied using groups: “optimal EN vs. non-optimal EN” in the 2014 and 2017 guidelines, respectively. EN was considered to be optimal if the cumulative enteral energy intakes were ≥90% of the recommended energy intakes for each guideline for at least half of the PICU stay, as used in the study of de Menezes at al. (10).

Quantitative variables were expressed as means (standard deviation) in the case of normal distribution or medians (interquartile range, IQR) otherwise. Normality of distributions was assessed using histograms and the Shapiro–Wilk test. Categorical variables were expressed as numbers (percentage). The association between the potential risk factors and the status regarding the application of the guidelines (non-optimal EN vs. optimal EN) was assessed using Student's *t* test or Mann–Whitney *U* test (according to the distribution) for continuous variables and chi-squared test for categorical variables. Variables with *p* < 0.1 in bivariate analysis were considered candidates for the multivariable model according to their clinical relevance as risk factors of the non-application of guidelines in the literature. These variables were included in a multivariable logistic regression. For each continuous predictor, the log-linearity assumption was assessed using the restricted cubic spline functions. The collinearity between the predictors was also examined with a maximum level for the variance inflation factor fixed at 2.5. Odds ratios for the risk of non-application of the guidelines were computed using 95% confidence intervals. Data were analyzed using the SAS software package, release 9.4 (SAS Institute, Cary, NC).

The study protocol was approved by the French Ethics and Legal committees (CER-SFP 2017-068, DEC20-220).

## Results

### Population Description

Of the 531 eligible patients, 416 were included (33, 47, 12, 11, six, and six patients were excluded according to criteria a, b, c, d, e, and f, respectively), with a median age of 14.5 months (IQR = 4–60). The median time taken to obtain the target energy intakes was 3 days (IQR = 1–4). The median time to initiate EN was 24 h (IQR = 7–48). Feeding intolerance occurred in 195 patients (47%) (Table 1).

**Table 1:** Demographic and clinical characteristics of the population.

**Variable**	***N* = 416**
Male patients, *n* (%)	226 (54.1)
Age, median month (IQR)	14.5 (4–60)
Weight, median kg (IQR)	9.1 (5.4–18)
PIM 2, median% (IQR)	2.2(1.1–6.8)
Chronic disease, *n* (%)	
Chronic cardiac disease	92 (22)
Chronic digestive disease	124 (29.7)
Malnutrition, *n* (%)	151 (36.2)
*Z*-score < −2	127 (30.5)
*Z*-score > 2	24 (5.7)
Diagnosis at PICU admission	
Respiratory	202 (48.5)
Neurologic	61 (14.6)
Surgical	59 (14.1)
Digestive disease	29 (6.9)
Cardiac disease	17 (4.1)
Sepsis	16 (3.8)
Burn	13 (3)
Trauma	12 (3)
Other	7 (1.6)
Mechanical ventilation, *n* (%)	201 (48)
Vasopressors, *n* (%)	53 (12)
Paralytic agents, *n* (%)	27 (6.4)
Morphine agents, maximum rate (mg kg^−1^ day^−1^), mean (range)	2.4 (6.5)
Midazolam, maximum rate (mcg kg^−1^ min^−1^), mean (range)	1.05 (2.55)
Procedures, *n* (%)	233 (55)
Time to initiate EN (h), median (IQR)	24 (7–48)
Enteral nutrition interruptions, mean (range)	1.1 (1.5)
Gastrointestinal intolerance, *n* (%)	195 (47)
Length of PICU stay (days), median (IQR)	5 (3–10)
Mortality at day 60, *n* (%)	34 (8.1)

*EN, enteral nutrition; IQR, interquartile; PIM 2, Pediatric Index of Mortality 2; PICU, pediatric intensive care unit*.

At PICU admission, 151 patients (36%) were malnourished: 127 underweight (30%) and 24 overweight (6%). Mortality rate at D60 was statistically increased in malnourished patients compared to normo-nourished patients (16 vs. 4%, *p* < 0.001), without differences in the median length of stay (5 vs. 6 days), PIM2 score (2.2% vs. 2.3%), and time to initiate EN (22 vs. 24 h).

### Comparison of Guidelines

The mean enteral energy intakes were 34 ± 30.3 kcal kg^−1^ day^−1^ and represented 45% of the recommended energy intakes for the 2014 guidelines and 66% for the 2017 guidelines according to each age classification (*p* = 0.03). The 2014 and 2017 guidelines were applied (>90% of the recommended energy intake at least 1 day) in 183 (44%) and 296 (71%) patients, respectively (*p* < 0.05). Following the 2017 American guidelines, enteral energy intakes were considered as “satisfactory enteral intake” (>60% recommended energy intakes at the end of the first week in PICU) for 335 patients (81%).

### Risk Factors of Non-application of Guidelines

Enteral nutrition was optimal (defined as cumulative enteral energy intakes ≥90% of the recommended energy intakes for each guideline for at least half of the PICU stay) for 88 patients (21%) for the 2014 guidelines and 161 patients (39%) for the 2017 guidelines (*p* = 0.003). For each guideline, there was no difference between optimal EN and non-optimal EN for 2014 and 2017, respectively, for mortality at D60 (*p* = 0.07 and *p* = 0.12), or length of stay (*p* = 0.16 and *p* = 0.81). For both guidelines, time to initiate EN was significantly longer in the non-optimal EN compared to the optimal EN group (24 vs. 10 h and 29 vs. 11 h, respectively; both *p* < 0.001).

In the multivariate analysis, hemodynamic failure was a risk factor of non-application of the guidelines for 2014 [odds ratio (OR) = 5.3, 95% confidence interval (CI) = 1.1–28.4] and 2017 (OR = 2.7, 95% CI = 1.1–7.5). Previous chronic cardiac disease was a protective factor of non-application of the guidelines for 2014 (OR = 0.5, 95% CI = 0.3–0.9) and 2017 (OR = 0.5, 95% CI = 0.3–0.9). For the 2017 guidelines, chronic digestive disease was a protective factor (OR = 0.6, 95% CI = 0.4–0.9) (Table 2).

**Table 2:** Univariate and multivariate analyses for optimal and non-optimal application of the 2014 and 2017 EN guidelines.

	**2014 French guidelines (*****n*** **=** **416)**					**2017 American guidelines (*****n*** **=** **416)**				
			**Univariate analysis^a^**	**Multivariate analysis^b^**				**Univariate analysis^a^**	**Multivariate analysis^b^**	
	**Optimal EN, *n* = 88 (21%)**	**Non-optimal EN, *n* = 328 (79%)**	***p***	**OR (95% CI)**	***p***	**Optimal EN, *n* = 161 (39%)**	**Non-optimal EN, *n* = 255 (61%)**	***p***	**OR (95% CI)**	***p***
PIM 2, median (IQR)	1.6 (0.8–4.5)	2.6 (1–6.7)	0.21			1.6 (0.8–4)	3.4 (1–7.4)	0.78		
Chronic cardiac disease, *n* (%)	32 (36)	60 (18)	<0.001	0.5 (0.3–0.9)	0.014	51 (32)	41 (16)	<0.001	0.5 (0.3–0.9)	0.019
Chronic digestive disease, *n* (%)	40 (45)	84 (25)	<0.001	0.6 (0.4–1.0)	0.075	67 (42)	57 (22)	<0.001	0.6 (0.4–0.9)	0.032
Malnutrition, *n* (%)	39 (44)	112 (34)	0.089	0.8 (0.5–1.3)	0.39	72 (45)	79 (31)	0.006	0.7 (0.4–1.0)	0.065
EN interruption, median (IQR)	0 (0–1)	1 (0–2)	0.12			0 (0–1)	1 (0–2)	0.004	1.1 (0.9–1.3)	0.41
Feeding intolerance, *n* (%)	44 (50)	151 (46)	0.48			74 (46)	120 (47)	0.83		
Invasive ventilation, *n* (%)	32 (36)	169 (51)	0.014	1.2 (0.7–2.0)	0.53	62 (38)	138 (54)	0.002	1.1 (0.7–1.8)	0.61
Non-invasive ventilation, *n* (%)	45 (51)	171 (52)	0.91			82 (51)	134 (53)	0.74		
Hemodynamic failure, *n* (%)	2 (2)	51 (15)	0.004	5.3 (1.1–28.4)	0.049	7 (4)	46 (18)	<0.001	2.7 (1.1–7.5)	0.047
Paralytic agents, *n* (%)	2 (2)	25 (8)	0.090	2.7 (0.6–12.9)	0.22	4 (2.5)	23 (9)	0.013	2.9 (0.9–9.1)	0.077
Morphinic agents, maximum rate (mg kg^−1^ day^−1^), median (IQR)	1.2 (0.6–2)	1.5 (1–6)	0.030	1 (0.9–1.1)	0.92	1.2 (0.7–2)	1.5 (1–7)	0.002	1.0 (0.9–1.1)	0.76
Midazolam, maximum rate (mcg kg^−1^ min^−1^), median (IQR)	1.7 (0.6–3)	1.5 (0.5–3)	0.65			1.5 (0.5–3)	1.5 (0.7–3)	0.67		
Outcomes										
Mortality at day 60, *n* (%)	1 (4)	31 (10)	0.071			9 (6)	25 (10)	0.12		
Length of stay in PICU (days), median (IQR)	6 (3–11)	5 (3–10)	0.16			5 (3–10)	5 (3–10)	0.81		
Energy intake received (kcal kg ^−1^day^−1^), median (IQR)	59.7 (41.8–73.5)	25.4 (12.7–38.4)	<0.001			52.4 (40.0–66.2)	18.4 (10.0–28.1)	<0.001		
Time to initiate EN (h), median (IQR)	10 (3–26)	24 (9–53)	<0.001			11 (4–27)	29 (12–58)	<0.001		

*EN, enteral nutrition; OR, odds ratio; CI, confidence interval; PICU, pediatric intensive care unit; IQR, interquartile range; PIM 2, Pediatric Index of Mortality 2*.

a *Mann–Whitney test for quantitative variables and chi-square for categorical variables*.

b *Multivariate logistic regression for the risk of non-optimal application of guidelines*.

## Discussion

This study is the first examining the applicability and risk factors of the non-application of two national guidelines in the PICU. In our 416 children (mortality rate, 8%), 36% of patients were malnourished at PICU admission. The mean enteral energy intakes (34 kcal kg^−1^ day^−1^) represented 45 and 66% of, respectively, the 2014 and 2017 guidelines' recommended energy intakes. The 2014 and 2017 guidelines were, respectively, applied to 44 and 71% of patients. Following the 2017 American guidelines, enteral energy intakes were considered as “satisfactory enteral intake” for 81% of patients. Hemodynamic failure was an independent risk factor of the non-application of both guidelines, whereas cardiac and digestive antecedents were protective factors.

Many studies have previously shown that the application of nutritional recommendations was difficult in the PICU, regardless of the previous guidelines used (10–12, 14). In the American prospective study by de Menezes et al., in the PICU (*n* = 207 children), only 20.8% of the population had energy intakes >90% of the target [estimated with World Health Organization (WHO) equations] (10). Moreover, Kyle et al. observed (*n* = 240 children) that the energy intakes were >90% of the energy intakes recommended by the Schofield equations for 40% of all patient-days in the PICU (11).

ESPHGAN guidelines provided a clinical guide of EN in pediatric patients (5), but basal metabolism was probably altered in critically ill patients and estimation of the optimal energy requirement was not provided (4, 15). The energy requirements differed between the two guidelines (Supplementary Table 1). The 2017 recommended energy intakes were lower than those of the 2014 guidelines (6, 7). The 2014 guidelines were based on the *Apports Nutritionnels Conseillés* reference, which is used in France to assess the nutritional status of the healthy population (8). The 2017 guidelines recommended energy intakes using the Schofield equations and were closer than the energy intakes measured by indirect calorimetry in the PICU (9, 16). In other words, the 2017 guidelines seem to be more accurate for establishing the nutritional targets for patients in the PICU. Several authors previously recommended a decrease in the target energy intakes, described as “autophagy,” in the acute phase in critically ill adults and children (14, 15, 17). Then, the 2017 guidelines recommended to target energy intakes equal to or >60% as estimated by predictive Schofield equations at the end of the first week in the PICU (7), closer than the recent adult recommendations (70% of the energy expenditure in the acute phase) (18). According to this threshold, “satisfactory enteral intake” occurred in 81% of the patients in our study.

In our study, hemodynamic failure was a risk factor of the non-application of both the 2014 and 2017 guidelines, as described in pediatric (WHO guidelines 1985, *n* = 84) (12) and adult (*n* = 2,410) patients (19). According to these studies, the definition of hemodynamic failure varies: vasopressor agents used, scoring system, or the lactate level increased (12, 20). Moreover, the 2017 adult nutritional guidelines recommend delaying EN when an uncontrolled shock with an increased lactate rate exists (21). In 2020, ESPNIC (European Society of Pediatric and Neonatal Intensive Care) suggested to start EN early in children who were stable on vasopressor agents (22).

In our study, chronic cardiac and digestive diseases were protective factors of the non-application of nutritional guidelines. As shown by de Menezes et al., malnutrition was more important in cases of chronic cardiac or digestive disease, and malnutrition was a protective factor of the non-application of the 2017 guidelines (10).

Our study had several limitations. Firstly, it was a single-center study. The population was heterogeneous (severity of illness, length of stay in the PICU, etc.), but comparable to those in previous pediatric studies (10, 11). Secondly, the applications of the guidelines were compared in a retrospective sample using a period of study at the beginning of the 2017 guidelines. However, the energy intake was probably somewhat independent of the guidelines because no EN protocol was used in our unit at that time. Thirdly, we did not compare the energy intake with the measured energy requirements in our study (indirect calorimetry or carbon dioxide production) (18) because these methods were not routinely used.

In our PICU, the received energy intake approached the level of intake recommended by the American 2017 guidelines, which used the predictive Schofield equations. They therefore seem more useful and applicable than the 2014 guidelines. Multicenter studies to validate the pediatric guidelines seem necessary.

## Data Availability Statement

The original contributions presented in the study are included in the article/Supplementary Material, further inquiries can be directed to the corresponding author/s.

## Ethics Statement

The studies involving human participants were reviewed and approved by French Ethics and Legal committees (CER-SFP 2017-068, DEC20-220). Written informed consent from the participants' legal guardian/next of kin was not required to participate in this study in accordance with the national legislation and the institutional requirements.

## Author Contributions

MJ and CG conceptualized the study, conducted the initial analyses, drafted the initial manuscript, reviewed, and revised the manuscript. FM performed the statistical analysis and revised the manuscript. J-BB analyzed and revised the manuscript. SL and MR conceptualized the study, reviewed, and revised the manuscript. AD performed statistical analysis and revised the manuscript. All authors have approved the final manuscript as submitted and agreed to be accountable for all aspects of the work.

## Conflict of Interest

The authors declare that the research was conducted in the absence of any commercial or financial relationships that could be construed as a potential conflict of interest.

## References

[1] De Souza Menezes F Leite HP Koch Nogueira PC. Malnutrition as an Independent Predictor of clinical outcome in critically ill children. Nutr Burbank Los Angel Cty Calif. (2012) 28:267–70. 10.1016/j.nut.2011.05.015 21872433

[2] Grippa RB Silva PS Barbosa E Bresolin NL Mehta NM Moreno YMF. Nutritional status as a predictor of duration of mechanical ventilation in critically ill children. Nutr Burbank Los Angel Cty Calif. (2017) 33:91–5. 10.1016/j.nut.2016.05.002 27364223

[3] Ventura JC Hauschild DB Barbosa E Bresolin NL Kawai K Mehta NM. Undernutrition at PICU admission is predictor of 60-day mortality and PICU length of stay in critically ill children. J Acad Nutr Diet. (2020) 120:219–29. 10.1016/j.jand.2019.06.250 31522971

[4] Wilson B Typpo K. Nutrition: a primary therapy in pediatric acute respiratory distress syndrome. Front Pediatr. (2016). 4:108. 10.3389/fped.2016.00108 27790606 PMC5061746

[5] Braegger C Decsi T Dias JA Hartman C Kolacek S Koletzko B. Practical approach to paediatric enteral nutrition: a comment by the ESPGHAN committee on nutrition. J Pediatr Gastroenterol Nutr. (2010). 51:110–22. 10.1097/MPG.0b013e3181d336d2 20453670

[6] Lefrant J-Y Hurel D Cano NJ Ichai C Preiser J-C Tamion F. [Guidelines for nutrition support in critically ill patient]. Ann Fr Anesth Reanim. (2014) 33:202–18. 10.1016/j.annfar.2014.01.008 24565944

[7] Mehta NM Skillman HE Irving SY Coss-Bu JA Vermilyea S Farrington EA. Guidelines for the provision and assessment of nutrition support therapy in the pediatric critically ill patient: society of critical care medicine and american society for parenteral and enteral nutrition. JPEN J Parenter Enteral Nutr. (2017) 41:706–42. 10.1177/0148607117711387 28686844

[8] Martin A. The 'apports nutritionnels conseillés (ANC)' for the French population. Reprod Nutr Dev. (2001) 41:119–28. 10.1051/rnd:2001100 11434516

[9] Vazquez Martinez JL Martinez-Romillo PD Diez Sebastian J Ruza Tarrio F. Predicted versus measured energy expenditure by continuous, online indirect calorimetry in ventilated, critically ill children during the early postinjury period. Pediatr Crit Care Med. (2004) 5:19–27. 10.1097/01.PCC.0000102224.98095.0A 14697104

[10] De Menezes FS Leite HP Nogueira PCK. What are the factors that influence the attainment of satisfactory energy intake in pediatric intensive care unit patients receiving enteral or parenteral nutrition? Nutr Burbank Los Angel Cty Calif. (2013) 29:76–80. 10.1016/j.nut.2012.04.003 22898265

[11] Kyle UG Jaimon N Coss-Bu JA. Nutrition support in critically ill children: underdelivery of energy and protein compared with current recommendations. J Acad Nutr Diet. (2012) 112:1987–92. 10.1016/j.jand.2012.07.038 23063414

[12] De Neef M Geukers VGM Dral A Lindeboom R Sauerwein HP Bos AP. Nutritional goals, prescription and delivery in a pediatric intensive care unit. Clin Nutr Edinb Scotl. (2008) 27:65–71. 10.1016/j.clnu.2007.10.013 18068875

[13] Mehta NM Corkins MR Lyman B Malone A Goday PS Carney LN. Defining pediatric malnutrition: a paradigm shift toward etiology-related definitions. JPEN J Parenter Enteral Nutr. (2013) 37:460–81. 10.1177/0148607113479972 23528324

[14] Mehta NM Bechard LJ Cahill N Wang M Day A Duggan CP. Nutritional practices and their relationship to clinical outcomes in critically ill children–an international multicenter cohort study^*^. Crit Care Med. (2012) 40:2204–11. 10.1097/CCM.0b013e31824e18a8 22564954 PMC3704225

[15] Joosten KFM Kerklaan D Verbruggen SCAT. Nutritional support and the role of the stress response in critically ill children. Curr Opin Clin Nutr Metab Care. (2016) 19:226–33. 10.1097/MCO.0000000000000268 26963579

[16] Meyer R Kulinskaya E Briassoulis G Taylor RM Cooper M Pathan N. The challenge of developing a new predictive formula to estimate energy requirements in ventilated critically ill children. Nutr Clin Pract. (2012) 27:669–76. 10.1177/0884533612448479 22677483

[17] Reintam Blaser A Berger MM. Early or late feeding after ICU admission? Nutrients. (2017) 9:1278. 10.3390/nu9121278 29168739 PMC5748729

[18] Singer P Blaser AR Berger MM Alhazzani W Calder PC Casaer MP. ESPEN guideline on clinical nutrition in the intensive care unit. Clin Nutr Edinb Scotl. (2019) 38:48–79. 10.1016/j.clnu.2018.08.037 30348463

[19] Reignier J Boisramé-Helms J Brisard L Lascarrou J-B Ait Hssain A Anguel N. Enteral versus parenteral early nutrition in ventilated adults with shock: a randomised, controlled, multicentre, open-label, parallel-group study (NUTRIREA-2). Lancet Lond Engl. (2018) 391:133–43. 10.1016/S0140-6736(17)32146-3 29128300

[20] Bakker J. Lactate levels and hemodynamic coherence in acute circulatory failure. Best Pract Res Clin Anaesthesiol. (2016) 30:523–30. 10.1016/j.bpa.2016.11.001 27931655

[21] Reintam Blaser A Starkopf J Alhazzani W Berger MM Casaer MP Deane AM. Early enteral nutrition in critically ill patients: ESICM clinical practice guidelines. Intensive Care Med. (2017) 43:380–98. 10.1007/s00134-016-4665-0 28168570 PMC5323492

[22] Tume LN Valla FV Joosten K Jotterand Chaparro C Latten L Marino LV. Nutritional support for children during critical illness: European Society of Pediatric and Neonatal Intensive Care (ESPNIC) metabolism, endocrine and nutrition section position statement and clinical recommendations. Intensive Care Med. (2020) 46:411–25. 10.1007/s00134-019-05922-5 32077997 PMC7067708

