# Supplementary material for: Are Nutritional Guidelines Followed in the Pediatric Intensive Care Unit?
Source: Front Pediatr. 2021 Jun 7;9:648867. doi: 10.3389/fped.2021.648867 (PMC8215211; doi:10.3389/fped.2021.648867)
Supplement: Supplementary file 1 [file Table_1.DOCX]

| Table 1. French Society of Anesthesia and Intensive Care Medicine (2014) and American Pediatric Nutrition group (2017) guidelines: Energy requirements | | | |
| --- | --- | --- | --- |
| Guidelines | Age | Energy requirements (Kcal/kg/day) | |
| French Society of Anesthesia and Intensive Care Medicine (2014) | 0-1 years* | 100-90 | |
|  | 1-6 years | 90-75 | |
|  | 7-12 years | 75-60 | |
|  | 13-18 years | 60-30 | |
|  |  |  |  |
| American Pediatric Nutrition group (2017) |  | Male | Female |
|  | <3 years | 59.512 xW -30.4 | 58.317xW-31.1 |
|  | 3-10 years | 22.706xW + 504.3 | 20.315xW + 485.9 |
|  | 10-18 years | 17.686xW +658.2 | 13.384xW +692.6 |
| **except new born, W= weight (kg)* | | | |
